# A Cutting Force and Hole Geometry Study for Precision Deep-Hole Microdrilling of Magnesium

**DOI:** 10.3390/mi15070814

**Published:** 2024-06-23

**Authors:** Margherita Pizzi, Antonio Costetti, Francesco De Gaetano, Marco Ferroni, Francesco Arleo, Federica Boschetti, Massimiliano Annoni

**Affiliations:** 1 Department of Mechanical Engineering, Politecnico di Milano, 20156 Milan, Italy; margherita.pizzi@polimi.it (M.P.); antonio.costetti@polimi.it (A.C.); massimiliano.annoni@polimi.it (M.A.); 2 Department of Chemistry, Materials and Chemical Engineering “Giulio Natta”, Politecnico di Milano, 20133 Milan, Italy; francesco.degaetano@polimi.it; 3 MgShell S.r.l., 20133 Milan, Italy; marco.ferroni@mgshell.com; 4 WatAJet S.r.l., 21010 Varese, Italy; francesco.arleo@watajet.com

**Keywords:** deep-hole microdrilling, magnesium, microholes, cutting forces, holes quality

## Abstract

Size effects, high thrust forces, limited heat dissipation, and tool deterioration are just some of the challenges that deep microdrilling poses, underscoring the importance of effective process control to ensure quality. In this paper, an investigation performed on a microdrilling process on pure magnesium using a 0.138 mm diameter microdrill to achieve an aspect ratio equal to 36 is proposed. The effect of the variation of the cutting parameters feed per tooth $f_{z}$ and cutting speed $v_{c}$ was studied on thrust force, supporting hole quality evaluation in terms of burr height, entrance, and inner diameters. The results showed that $f_{z}$ significantly influences the hole quality. In fact, as $f_{z}$ increases, the burr height decreases and the inner diameter approaches the nominal diameter. However, optimizing the hole geometry with high feed per tooth values increases the thrust forces, compromising tool life. In fact, a significant dependence of the thrust force on both cutting parameters was found. In this scenario, increasing $v_{c}$ can mitigate the high thrust forces by inducing material softening. The study results improve precision manufacturing by refining parameters, ensuring the quality and reliability of magnesium-based microcomponents.

## 1. Introduction

Deep microdrilling is a very complex manufacturing process, typically employed when there is a need for precise control over hole diameter, coupled with a requirement for excellent straightness and surface finish. It is usually referred to as deep drilling when the hole depth *D* is 10 times larger than the nominal hole diameter *d* [1,2]. Therefore, the aspect ratio (AR) *D/d* > 10 is defined. Here, limited heat dissipation, intricate chip formation, and high trust force values are attributable to the depth of the cutting zone within the workpiece [3,4]. These phenomena are further emphasized in the micro domain by the small size of the tools, whose cutting edges have a radius comparable to the grain size of the material being cut, thus, size effects occur [5]. Microdrills with a higher AR have a higher ratio of flute length to drill bit diameter. This contributes to lower rigidity and a higher risk of bending fracture [6]. In addition, this micromanufacturing process is subject to various constraints, including susceptibility to vibration and rapid deterioration of cutting tools, requiring frequent tool changes to reduce errors in straightness, roundness, and cylindricity of microholes [5,7,8]. For this reason, in areas such as aerospace or biomedicine, where deep microdrilling is widely used for its precision, measuring the diameter and depth of microholes and monitoring their internal geometry quality is essential. However, conventional measuring tools may have limitations in reaching the bottom of these holes or providing accurate measurements, causing uncertainties in hole quality assessment when high ARs take place. In fact, as depth increases, maintaining the diameter accuracy over the entire length of the hole becomes more difficult. Typically, destructive methods are used to evaluate the internal shape of microholes, which includes cutting cross-sections of holes and/or creating plastic replicas to reproduce the microholes [9]. In fact, in these cases, conventional coordinate measuring machines (CMMs) and vision-based systems are unable to perform shape measurements on deep microholes [10,11]. For example, in the study conducted by Diver et al. [12], the quality of reverse tapered microholes made by means of electrical discharge machining is examined using a new 3D imprint technique. This involved the use of hole impressions, and thus, sectioning the samples.

In light of this, the need to monitor the process in such a way as to ensure maximum accuracy in microcomponents without the need to section the machined parts arises. Generally, monitoring the micromilling or microdrilling process is critical to obtaining accurate parts. This process provides insight into the cutting mechanism and the occurring phenomena such as chip formation, vibration, and tool wear [13,14,15,16]. Monitoring can involve the use of accelerometers, force or torque sensors, and current sensors. The data obtained, therefore, can be used as a diagnostic tool during machining [17]. Thus, although continuous monitoring and control of the drilling process is essential to maintain the desired quality, real-time monitoring of deep-drilling microholes can be difficult. Implementing effective control systems is crucial to detect deviations and adjust process parameters accordingly. The relationship between cutting forces during deep microdrilling and hole morphology could provide insight into material removal mechanisms and challenges associated with the microscale [18,19]. The interaction between the cutting tool and the workpiece provides information about the cutting mechanism, specific interaction with the tool, and machine and tool conditions (such as wear, geometric runout, and vibration). The modulus and directions of the x- and y-components of the cutting force may contain relevant data on runout, consequently, the possible presence of geometric errors can be detected. This information is a valuable resource for the development and implementation of monitoring and diagnostic techniques in the machining process, especially when the small size does not allow the use of contact measurements. Kim et al. [20] developed a method to improve the durability of microdrills in deep drilling on steel. The tests showed that the tool breakage event was preceded by a gradual increase in thrust force, spindle speed harmonic component and frequency content amplitudes in the 0–100 Hz range. Monitoring parameters of the process were finally chosen as the slope of the average thrust force signal, the peak-to-valley value of low-pass and high-pass filtered thrust forces.

An experimental campaign to study the effect of feed and cutting speed on cutting force components and hole quality in carbon fiber reinforced plastic composite (CFRP) material is described in the study conducted by Anand et al. [21]. From this type of approach, it became possible to derive fundamental information for obtaining good hole quality in CFRP materials. It was observed that the cutting forces and hole quality are influenced by size effects in microdrilling. Specifically, the lowest cutting forces and the lowest hole quality error were obtained with feed close to the tool cutting edge radius, contrary to what was expected.

In light of what is present in the state of the art, this article reports a study performed on a microdrilling process on pure magnesium using a 0.138 mm diameter drill bit to achieve an AR equal to 36. Microdrilling experiments were performed to study the effect of feed per tooth and cutting speed on cutting forces, particularly thrust force, to support the study on hole quality as a function of cutting parameters. Hole quality was evaluated in terms of burr height, entrance diameter, and inner diameter.

The choice of magnesium as the material for this experimental study is well justified, especially in the biomedical field, where implantable devices must function for a limited period. Corrosion within body fluids allows the device to dissolve completely once its function is achieved, eliminating the necessity for surgical removal. Indeed, being biocompatible and biodegradable, this material is very suitable for the development of (i) drug delivery devices, (ii) needles, and (iii) implants for orthopedic applications [22,23,24,25]. The goal of this work is to bring innovative results to the state of the art on magnesium deep microdrilling, leading to a better understanding of the mechanisms of material removal in a difficult-to-monitor process. In fact, there are no studies in which the chip removal mechanism of pure magnesium with sub-millimeter diameter tools with such high AR is investigated. The study conducted by Sun et al. [26], for example, proposes an experimental campaign on magnesium using drills of 1 mm in diameter and 9.5 mm in length (AR = 9.5), by drilling holes 4 mm deep. In the study conducted previously by the same authors of this paper [27], the effect of cutting parameters on the quality of holes with a diameter 0.200 mm and depth about 4 mm (AR = 20) was investigated. In the present study, it is intended to increase the aspect ratio by almost two times, decreasing the tool diameter by 31%, and increasing the hole depth by 25%. This makes machining more risky due to (i) lower tool rigidity [6], (ii) phenomena associated with microscaling such as minimum chip thickness [5,28], and (iii) greater difficulty in chip evacuation from hole depth [3]. In addition, the analysis of the forces developed during the process and the analysis of the internal geometry of the hole are introduced.

## 2. Materials and Methods

### 2.1. Workpiece, Tools and Strategy

**Figure 1:**
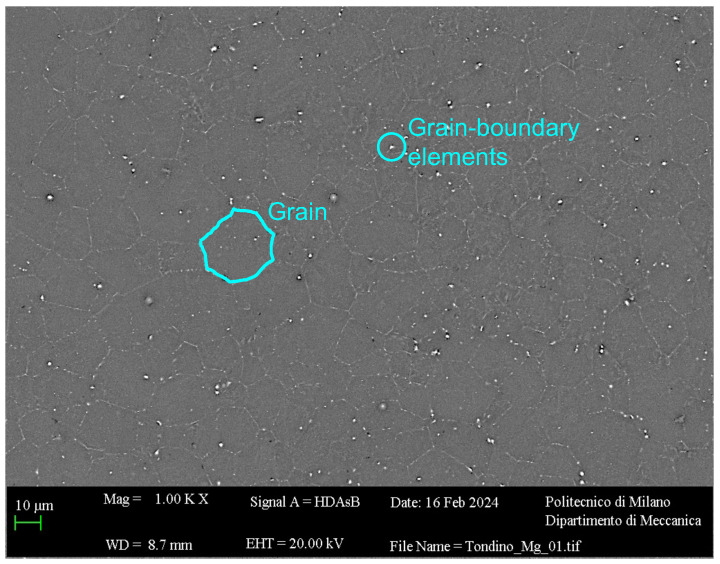
Sample micrography with grain and grain-boundary elements.

The experiments were performed on pure magnesium rods (diameter $D_{\mathrm{rod}}$ = 20 mm, thickness *th* = 10 mm), whose mechanical properties and composition were evaluated in a previous study and are reported in Table 1 [27]. The average grain size $\bar{d}$ = 16.1 µm (σ = 1.67 µm) was measured from the image shown in Figure 1, obtained by means of a Scanning Electron Microscope (SEM, Zeiss EVO 50XVP, Carl Zeiss, Oberkochen, Germany) [27].

Magnesium is usually characterized by good machinability thanks to its favorable mechanical properties [29], such as low hardness. However, in deep-hole drilling the morphology of the chip is usually long and forced into a confined region, thus is more difficult to wash away by the oil stream. In this condition, magnesium machining is even more challenging: the heat generated during drilling can cause a reduction in its hardness, promoting the adhesion of softened material to the drill bit flutes. This results in the formation of a large built-up edge on the tool tip [30]. This means that this metal tends to deform during drilling rather than form clean chips, leading to poor surface finish and tool wear.

The fabrication of microholes characterized by high AR requires very precise machines to ensure the accuracy of the final part. For this reason, the Kern EVO (Kern Microtechnik GmbH, Eschenlohe/Murnau, Germany), an ultra-precise five-axis machining center whose accuracy on the part is in the ±2.0 µm range, was used. This machine is equipped with a spindle capable of rotating in a speed range of 500 to 50,000 rpm, delivering a maximum power of 6.4 kW. Due to the high speed the spindle can reach, acceptable cutting speeds can be achieved when tool diameters decrease.

**Figure 2:**
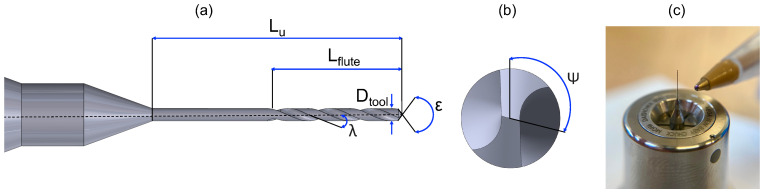
Microtwist drill geometry. (**a**) Longitudinal view. $D_{\mathrm{tool}}$: tool diameter; $L_{\mathrm{flute}}$: flute length; $L_{u}$: tool usable length; ϵ: point angle; λ: helix angle. (**b**) Front view. ψ: chisel edge angle. (**c**) 0.138 mm microdrill.

**Figure 3:**
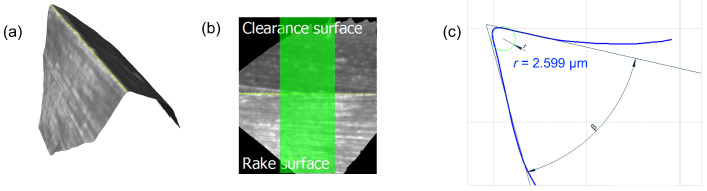
Cutting edge radius measurement by means of Alicona InfiniteFocus G6. (**a**) Acquisition of the cutting edge with the EdgeMasterModule. (**b**) Portion of the cutting edge highlighted in green containing the 800 profiles to be averaged. (**c**) The averaged profile of the cutting edge was evaluated within the region of interest.

A centering operation was conducted prior to deep drilling to enhance the stability of the microdrill during the subsequent process [31]. In this case, a 0.140 mm diameter pilot drill (Louis Belét, Vendlincourt, Switzerland) was employed. Then, deep microdrilling was performed, with a two-cutter drill bit (Louis Belét) with a diameter of 0.138 mm and usable length of 5.5 mm. A drawing showing the geometry of this tool is shown in Figure 2. The cutting edge’s radius ($r_{e}$) for the 0.138 mm microdrill was determined through measurements conducted using the 3D microscope Alicona InfiniteFocus G6 (Bruker Alicona, Graz, Austria). To obtain this value, the appropriate EdgeMasterModule was employed, providing direct insights into the tool’s geometrical features (see Figure 3). The cutting edge radius of the pilot drill was not measured, as it is not significantly involved in the process of interest of deep drilling. The properties of the tools are shown in Table 2. Before conducting the experiments, the tools were measured using the Marposs® Visual Tool Setter (VTS, Marposs S.p.A., Bologna, Italy) directly on the Kern EVO. Specifically, runout was measured as the total indicator reading (TIR) at the operating speed, which represents a phenomenon in which the effective diameter of the tool widens during rotation. In fact, rotation, coupled with an imbalance within the tool holder-tool system, results in the generation of centrifugal force and thus runout. The adequacy of the tool mounting in the tool holder was acceptable when the TIR was less than 4 µm, because otherwise the probability of drill breakage during the early stages of drilling is very high.

**Figure 4:**
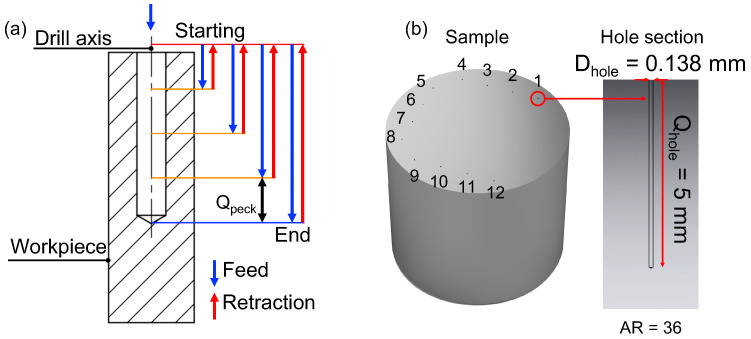
(**a**) Deep-drilling strategy with retraction of the tool. $Q_{\mathrm{peck}}$ represents the peck depth. (**b**) Sample with hole run order and hole section.

Peck drilling with tool retraction was chosen as the strategy to promote heat dissipation, avoid chip adhesion, and facilitate chip evacuation (see scheme in Figure 4a) both for the pilot and deep-drilling operations [6,20,32]. To improve cutting conditions [33], a lubricant (Blasogrind HC5) with a very light flow was also supplied so as not to destabilize the microdrill during the process. In Figure 4b a representative CAD of the drilling pattern is shown and an example of the section of one of the holes is reported. A 2^2^ factorial design with three replicates, i.e., 12 holes, was chosen for the experiment, allowing for the variation of factors $f_{z}$ and $v_{c}$ at two levels each.

**Figure 5:**
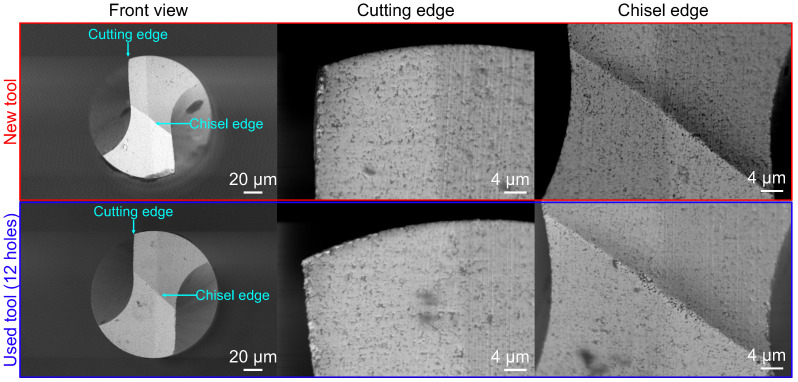
A 0.138 mm drill observed with SEM. From the front view, the cutting edge and chisel edge of a new tool are compared with those of the tool that has been used to make 12 holes: no significative wear is found.

In this study, the effect of tool wear was considered as a systematic external factor affecting the measurements, which, according to the Design of Experiments (DoE) procedure, is minimized by randomization of the tests [34]. In addition, the drill bit was compared before and after the experiments by SEM. Figure 5 shows that wear is imperceptible in the tool used for the experiment: both the chisel edge and the cutting edges appear as sharp as those of the new tool. Therefore, a single tool was used for all 12 holes, and the individual tests were randomized in order to average out the effects of external factors and tool wear. The ranges of feed per tooth and cutting speed were determined in accordance with the specifications provided by the manufacturers of the respective tools and preliminary tests. All cutting conditions were reported in Table 3 and the executed experimental drilling plan for the 0.138 mm holes in Table 4.

The choice of restricted ranges for cutting parameters in micromachining derives from two main reasons. First, the main constraint is dictated by the feed per tooth: too low $f_{z}$ leads to the ploughing condition and thus to drill bit breakage, while too high $f_{z}$ can lead to tool breakage because of buckling. Second, the signal analysis during experimentation revealed vibrations due to spindle rotation between 30,000 rpm and 45,000 rpm (in accordance with the manufacturer’s recommended parameters for magnesium), complicating the interpretation of the force signal. To mitigate this problem, rotational speeds and thus cutting speeds $v_{c}$ were chosen to minimize vibration in all directions. Consequently, two levels of parameter variation seemed suitable given the constraints of the machining process and the machine.

### 2.2. Acquisition and Eleboration of Force Signals

#### 2.2.1. Data Collection Hardware

**Figure 6:**
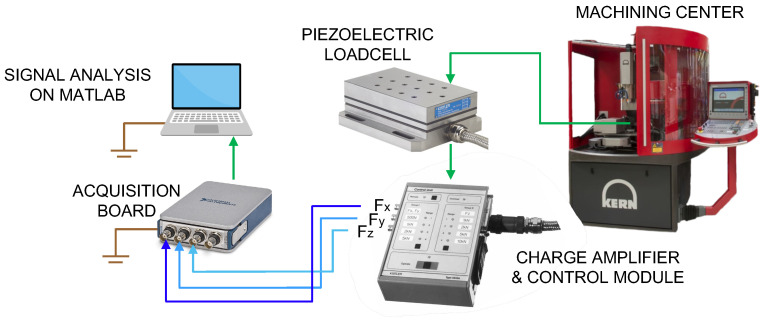
Cutting force acquisition system.

In this work, a multi-component piezoelectric dynamometer, Kistler Type 9257BA (Kistler Group, Winterthur, Switzerland), was used to monitor the process, measuring the three orthogonal force components *F_x_*, *F_y_* and *F_z_* with a 51,200 Hz sampling frequency. Being based on the piezoelectric operating principle, this dynamometer is suitable for measuring even low variations and values of forces, as expected during micromachining. The dynamometer comprises four three-component force sensors installed under high preload between a base plate and a cover plate. These force sensors measure the force components directly, with minimal displacement. The dynamometer incorporates a built-in three-channel charge amplifier, resulting in a low-impedance output signal. The load cell, equipped with a test specimen, was installed inside the Kern EVO machining center. The signals from the Kistler Type 9257BA piezoelectric transducer are thus processed by the Type 5233A1 Kistler control unit. In this work, a full scale of 1 kN was selected for signals in x, y, and z directions. Then, the outputs in the three directions are acquired by a four-channel NI9234 USB acquisition board (National Instruments, Austin, TX, USA) and sent to a computer for analysis using Matlab (R2020a, MathWorks, Natick, MA, USA). This process and the equipment are represented in Figure 6. Every acquisition session was preceded, as suggested by the user manual, by a minimum of 60 min warm-up to ensure thermal equilibrium.

#### 2.2.2. Signal Processing

Before applying the Fast Fourier Transform (FFT), the acquired signals were windowed to avoid the presence of discontinuities in its periodized representation, thus leading to a more accurate representation of their frequency content. In this study, the Hann window was chosen, which is among the most widely used windows due to its satisfactory trade-off between sharpness and attenuation [35].

These signals were first subjected to a filtering operation (during analog-to-digital conversion) to reject the frequency band affected by aliasing. Despite the large alias-free frequency content of the raw signal, due to the complexity of the microdrill–material interaction, an additional cutoff filter was applied with a cutoff frequency $f_{\mathrm{cutoff}\_n}$ associated with rotational speed as reported in Equation (1):

(1) $$f_{\mathrm{cutoff}\_n}=n\cdot \frac{Z}{60}$$

where *n* is the rotational speed in [rpm] and *Z* is the number of cutting tool teeth. In fact, the multitude of phenomena related to the cutting action (drill bit–material interactions or chip–flute interactions) added to the dynamic microdrill–workpiece behavior, making the presence of high-frequency contributions extremely likely.

**Figure 7:**
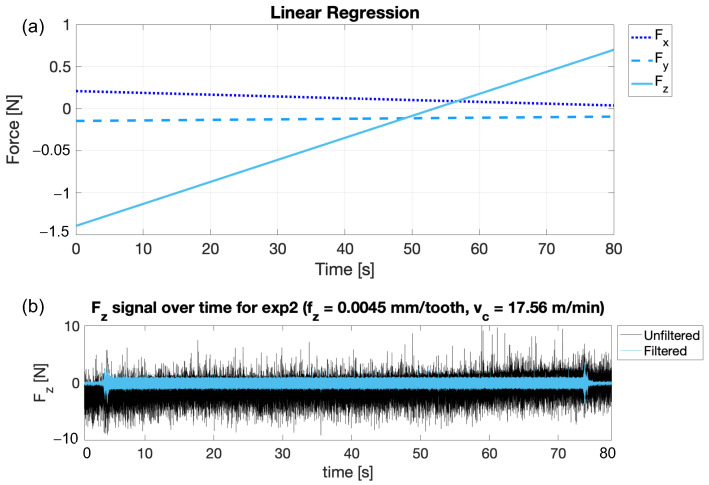
(**a**) Linear regression of the force signals for Run 02 (exp2) in the x, y, and z directions showing the drift effect. (**b**) Run 02 (exp2) filtered force signal in the z direction, compared with the raw one affected by drift.

Since drift was observed in some signals, especially in the z direction, an additional high-pass filter was applied. Linear regression of the force signals for Run 02 (exp2) in the x, y, and z directions showing the drift effect is reported in Figure 7a. The same filtered force signal in the z direction, compared with the raw one affected by drift, is shown in Figure 7b. Looking at the extent of the trends, the sensor piezoelectric drift was excluded as the main cause, as it is claimed by the manufacturer to be ≤±0.01 N/s along the z direction, and ≤±0.005 N/s along x and y. Indeed, an increase of 0.26 N ca. on *F_z_* can be appreciated over a period of 10 s, which is greater than the estimated 0.1 N of piezoelectric drift. Therefore, the piezoelectric effect is excluded from the possible causes.

**Figure 8:**
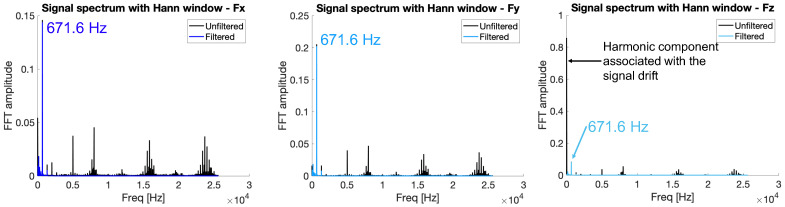
Signal spectrum for FFT of Run 02 (exp2). Each graph shows the frequency content of the force signals in the x, y, and z directions that have been processed. These show how, after the filtering operation, nonsignificant frequencies unrelated to the spindle rotation frequency were removed without altering the amplitude in a relevant way.

**Figure 9:**
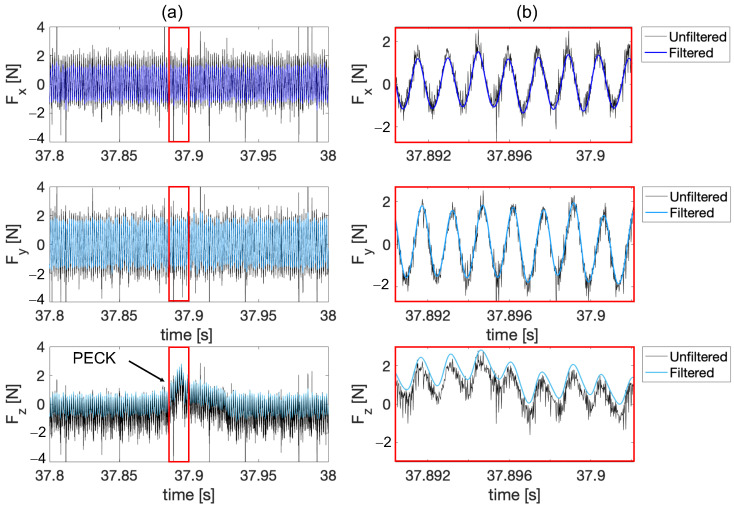
(**a**) Force signals in the x, y, and z directions superimposed with the raw signals for Run 02 (exp2). (**b**) Close up of the signal where the sinusoidal behavior can be observed during the cutting phase (peck region).

Another plausible cause that can be attributed to the signal drift is the thermal effect caused by the stream of lubricant oil that covers the specimens during the operation, and that falls onto the loadcell acting as a heat source. In order to remove this effect, a high-pass filter with $f_{\mathrm{cutoff}\_\mathrm{drift}}$ = 1 Hz was applied. The decision to use a frequency of 1 Hz was based on tests performed to mitigate the piezoelectric drift effect of the sensor while avoiding excessive signal attenuation. Choosing a too low frequency (i.e., 0.01 Hz) would not adequately imply reduced drift, while choosing a too high frequency (i.e., 10 Hz) would have resulted in unacceptable signal loss. In fact, tests in these condition showed that the amplitude of $F_{z}$ during a peck for the straightened signal and the one affected by drift were comparable with deviation in the range of ± 0.02 N. In this case, the amplitude was computed as the difference between the maximum value and average value of the baseline vibration, i.e., the harmonic signal present all across the operation, synchronous with the spindle speed, and associated with the worktable vibration as a result of the excitation introduced by the spindle imbalance. For this reason, the pass-band filtration was then carried on in the processing of all data from the experimental campaign. Figure 8 shows the frequency content obtained with the Fourier transform (FFT) for the unfiltered and filtered signals of the forces in the three directions for Run 02 (exp2). The filtering produces a significant reduction in the harmonic component associated with the signal drift, which in the case of *F_z_* represents the dominant component of the spectrum in the raw signal and almost disappears in the filtered one. Moreover, the spindle speed harmonic component, namely 671 Hz, remains nearly unaffected by the filtering procedure. Figure 9 shows a part of the signal (Run 02—exp2) of the forces in the x, y, and z directions when one single peck occurs. Here, both filtered signals superimposed on raw signals can be observed. In particular, it is possible to say that the filtered signal consistently follows the sinusoid of the unfiltered one, with no background noise.

#### 2.2.3. Signal Analysis

Since material removal occurs only in some intervals according to the used peck drilling strategy, it was necessary to isolate the individual pecks in the acquired signals. To do this, an algorithm was developed in Matlab. The main idea of the Matlab code dedicated to signal analysis is to extrapolate the three cutting force components using the signal of the thrust force *F_z_* as a trigger for the occurrence of the cutting actions. Indeed, a noticeable spike occurs whenever the engagement of the cutting edges takes place (see the close up of the force signal in the z direction in Figure 9b). Therefore, a tunable and parametric algorithm capable of identifying and isolating, with minimal user input, every spike associated with the cutting action and avoid any spikes of a random nature was written.

**Figure 10:**
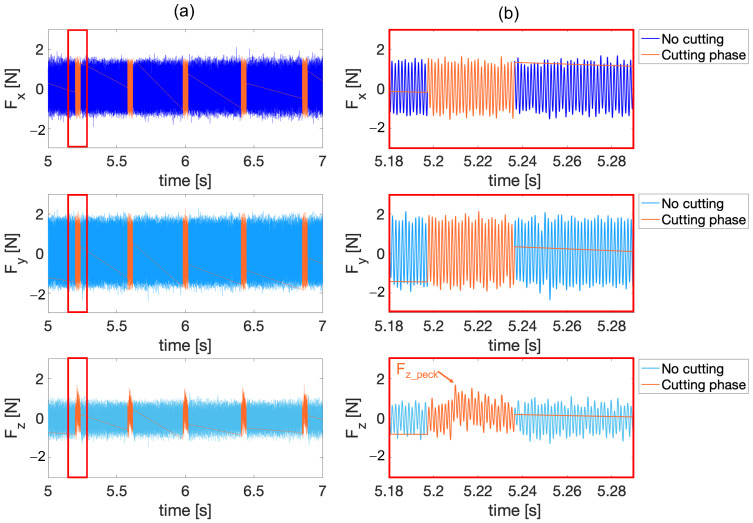
Filtered signals of the *F_x_*, *F_y_* and *F_z_* forces for Run 02 (exp2) with peck isolation. In the no cutting regions material removal does not take place because the tool is repositioning into the hole during peck drilling. (**a**) First five pecks of the drilling process. (**b**) Close up of the first peck. Here, the maximum value detected in the cutting phase (one single peck) $F_{z\_\mathrm{peck}}$ is shown.

At the peck-related time window identified in *F_z_*, the time windows of *F_x_* and *F_y_* were also extracted. This procedure is shown in Figure 10a (Run 02—exp2). Here, the pure cutting phases, in which material removal takes place, were isolated from the rest of the signal, in which the tool is repositioning and therefore not cutting. Here, the maximum value detected in the cutting phase (one single peck) $F_{z\_\mathrm{peck}}$ is shown.

However, extraction of the values of the forces in the x and y directions associated with the cutting phase showed no significant changes in the amplitude or change in the mean value in either direction. This is undoubtedly related to the asymmetries of the microtools, which, being submicrometric, generate minimal imbalances in the forces [20]. In fact, as can be deduced from the signals shown in Figure 10b, the magnitude of the runout, if existing, is so small that no significant difference in the forces are observed with respect to the background vibration of the machine table of 1 N amplitude.

Moreover, it can be seen from Figure 9 and Figure 10 that, due to the vibration mode of the machine, the uniform vibration over time in x and y in the cutting and no cutting phases are greater than in the z direction, where the peck phase is well distinguishable. If the amplitude of force signals in the x and y directions is affected by machine vibration, it is natural to infer that the amplitude of force signals in the z direction should also be altered by the same phenomenon. For this reason, in this paper, an analysis is not made on the absolute value of the forces in the z direction, rather the trend is evaluated as a function of the peaks during deep-drilling machining. This choice is justified as the regions of material removal are well distinguishable within the signals. For this reason, only the thrust force *F_z_* will be taken into consideration. Specifically, the maximum force values (one per peck, sixty-six in total for a run) $F_{z\_\mathrm{peck}}$ were collected for each run i.e., each hole. Thus, the maximum value among $F_{z\_\mathrm{peck}}$ and the average value of all $F_{z\_\mathrm{peck}}$, $F_{z\_\max}$ and $F_{z\_\mathrm{avg}}$ respectively, were analyzed.

### 2.3. Holes Quality Analysis

To evaluate hole quality as a function of cutting parameters, burr height, entrance diameter, and diameter along the axis of the blind hole were measured in this study. Obviously, surface imperfections such as burrs must be minimized when dealing with miniaturized devices where high-part precision is required. Also, in this context it is essential to check the involved tolerances, which must remain within a very tight range.

#### 2.3.1. Burr Height and Entrance Diameter

**Figure 11:**
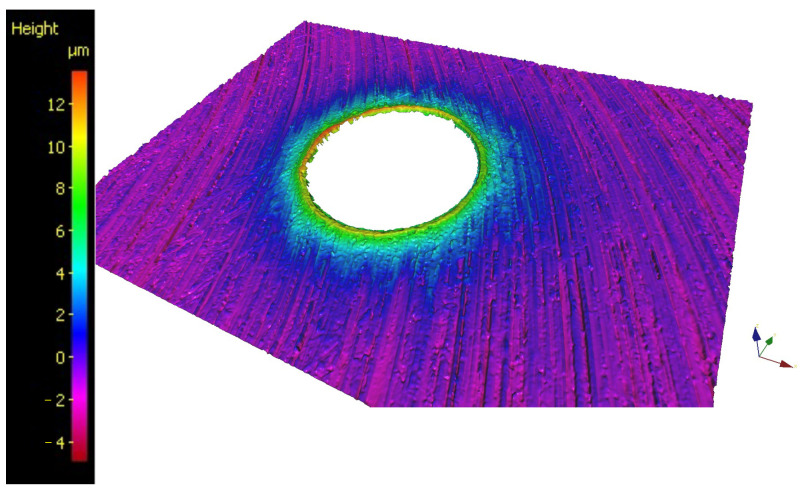
3D dataset of the hole entrance, from which the burr height and the hole entrance diameter were measured.

The 3D dataset used to measure burr height *H*_burr_ and hole entrance diameter *D*_entr_, shown in Figure 11, was acquired using the AliconaG4 3D optical microscope (Bruker Alicona, Graz, Austria). Specifically, through the IP-LaboratoryMeasurementModule 5.1 software, an objective with X50 magnification and a measurement range of 250 × 220 µm was used. After the surface acquisition, the dataset coordinates reference system was adjusted by fitting a plane to correctly identify the z-axis orientation. The “best-fit function” was chosen for the fitting procedure.

**Figure 12:**
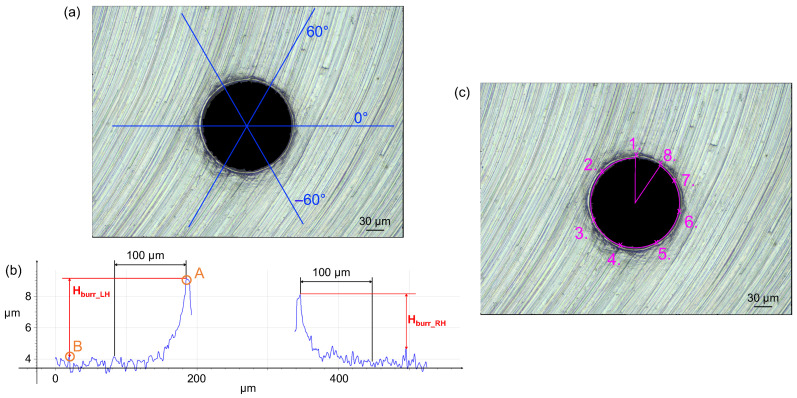
*H*_burr_ and *D*_entr_ measurement. (**a**) Selected profiles for burr height measurement. (**b**) Example of burr profile. A and B are the selected points to measure the burr height. *H*_burr_LH_: left burr height; *H*_burr_RH_: right burr height. (**c**) Entrance diameter *D*_entr_ measurement.

The procedure described in BS EN ISO 8785:1999 [36] was followed to measure the burr height. In this case, the ProfileFormMeasurement function of the Alicona software (MeasureSuite 5.3.9, LaboratoryMeasurementModule 5.1) was used to analyze the surface profile selected on the 3D dataset. A total of three lines were drawn through the center of the circumference of the borehole at angular positions 0°, 60°, and −60°, as shown in Figure 12a. For each profile, two burr heights were measured: one on the right (*H*_burr_RH_) and one on the left (*H*_burr_LH_). These were measured as the distance between the maximum peak of the burr profile (point A) and the highest point at a minimum distance of 100 µm from A (point B). This procedure is reported in Figure 12b. As a result, six burr height measurements were obtained for each hole.

The diameter of the entrance hole was measured using the 2DImageMeasurements Alicona software tool (MeasureSuite 5.3.9, LaboratoryMeasurementModule 5.1), which allows direct measurements on the 2D image. The diameter was reconstructed by fitting a circle on eight manually selected points along the circumference of the entrance hole, as observed in Figure 12c. The measurement was repeated three times for each hole.

#### 2.3.2. Diameter along Hole Depth

In order to measure the inner diameter and reconstruct the internal geometry of the hole, the Alicona InfiniteFocusG6 3D microscope (Bruker Alicona, Graz, Austria) was used. Specifically, the VerticalFocusProbing tool (MetMax 3.5 and LaboratoryMeasurementModule 10.5), which allows precise and repeatable high-resolution measurements of slope angles greater than 90°, was used. The measurement uncertainty on distances in the x and y directions is declared to be equal to 0.7 µm for distances below 1 mm. As a result, evaluation of internal geometries and microholes is possible. The limitation, again, is associated with the high AR of the holes. In fact, the instrument manufacturer states the possibility of acquiring microholes for AR approximately equal to 10, which is much less than in the present case (AR = 36).

**Figure 13:**
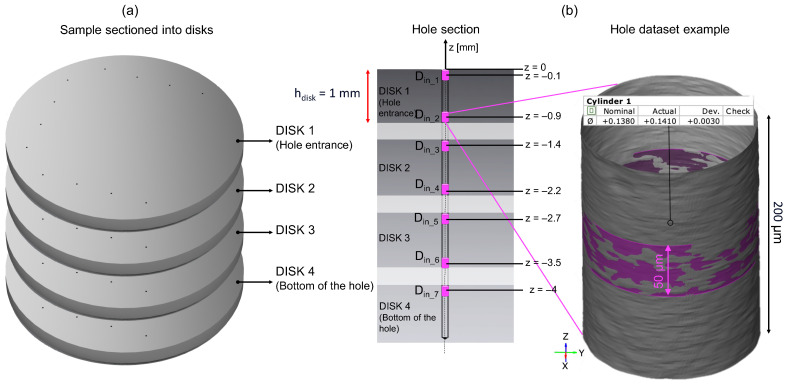
(**a**) Scheme of the WEDM-sectioned specimen for the use of Vertical Focus Probing of Bruker Alicona. (**b**) Hole section and hole 3D dataset. The seven measurements along the hole axis are reported and named from *D*_in_1_ to *D*_in_7_. Measurements are obtained by fitting a cylinder in the 3D dataset on GOM Inspect.

For this reason, the specimen was sectioned by wire electrical discharge machining (WEDM) transversely into 1 mm-thick disks (see Figure 13a). After some preliminary tests to assess the maximum acquisition depth achievable while ensuring satisfactory measurement quality (number of points), it was decided to acquire a depth of 200 µm from the borehole entrance on both sides of the disks. Deeper acquisitions in fact caused a gradual and rapid reduction in the density of the point cloud, which means a less accurate dataset. Therefore, the measurement of the diameters along the depth of holes was carried out using the GOM Inspect software (version 2.0.1, Carl Zeiss, Oberkochen, Germany). To maintain consistency in the diameter measurement of each dataset, a 50 µm height region of interest (purple region in Figure 13b) was selected at the center of the 200 µm acquisition to best fit the cylinder in all hole datasets. This allowed us to obtain the same number of points for all measurements, since some datasets had disturbances in some regions that did not allow the entire point cloud to be used. The measurement points from *D*_in_1_ to *D*_in_7_ were positioned at z = −0.1, −0.9, −1.4, −2.2, −2.7, −3.5, and −4.0 mm from the entrance hole. The trend of the hole diameter along its axis from the seven measurements (Figure 13) was evaluated (see Section 3.2).

## 3. Results

The statistical analysis of the acquired data was carried out using Minitab 21 (Minitab, Ltd., Coventry, UK). Once the normality of the data was verified, a Two Way ANOVA (TWA) was performed to test the effect of the cutting parameters and their interaction. If normality was not verified, the nonparametric Kruskall–Wallis (KW) test was used to check the effect of $v_{c}$ and $f_{z}$.

### 3.1. Cutting Forces

**Figure 14:**
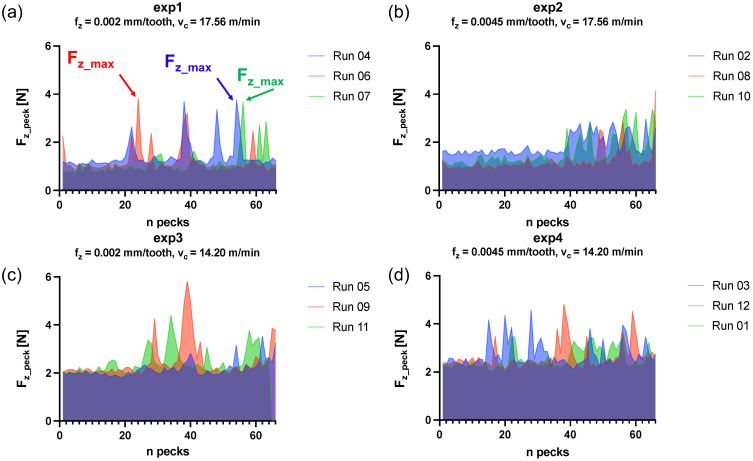
(**a**) $F_{z\_\mathrm{peck}}$ for every peck for the 3 runs of exp1. $F_{z\_\max}$ of each run is reported as example. (**b**) $F_{z\_\mathrm{peck}}$ for every peck for the 3 runs of exp2. (**c**) $F_{z\_\mathrm{peck}}$ for every peck for the 3 runs of exp3. (**d**) $F_{z\_\mathrm{peck}}$ for every peck for the 3 runs of exp4.

**Figure 15:**
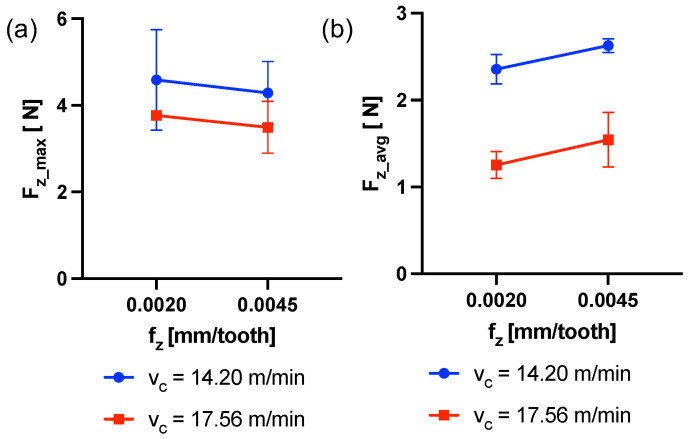
(**a**) Interaction plot of $f_{z}$ and $v_{c}$ on $F_{z\_\max}$. (**b**) Interaction plot of $f_{z}$ and $v_{c}$ on $F_{z\_\mathrm{avg}}$.

In Figure 14, the trend of the maximum value for each peck $F_{z\_\mathrm{peck}}$ is shown grouped by experiment (Table 4). Regions of constant $F_{z\_\mathrm{peck}}$ can be identified and associated with a stable cutting condition with good chip extraction from the cutting area. Other regions exhibit much higher variability, which could be associated with unstable conditions due to a critical chip evacuation. Among all experiments, exp2 ($f_{z}$ = 0.0045 mm/tooth, $v_{c}$ = 17.56 m/min) showed the most stable cutting condition until the half of the drilling process (37–38 pecks), after which the thrust force increases (Figure 14b). In contrast, exp4 ($f_{z}$ = 0.0045 mm/tooth, $v_{c}$ = 14.20 m/min) revealed a more unstable cutting condition throughout the entire process, suggesting that its combination of parameters is more prone to chip clogging (Figure 14d). The maximum force $F_{z\_\max}$ and the average force in the z direction $F_{z\_\mathrm{avg}}$ were considered. The former is the absolute maximum value of thrust force (Figure 14a), the latter was measured as the average of the maxima of all pecks for each run. Statistical analysis was conducted by the TWA for $F_{z\_\max}$. In this case, no statistically significant dependencies emerged from the cutting parameters or their interaction (*p*-Value > 0.05). The results are plotted in Figure 15a. The TWA test showed a statistically significant dependence of the response $F_{z\_\mathrm{avg}}$ on both parameters but not on their interaction (*p*-Value ($f_{z}$) = 0.04, *p*-Value ($v_{c}$) < 0.001, *p*-Value ($f_{z}$$v_{c}$) > 0.05). Figure 15b shows how the average thrust force increases with increasing feed per tooth and decreases with increasing cutting speed.

### 3.2. Holes Quality Results

**Figure 16:**
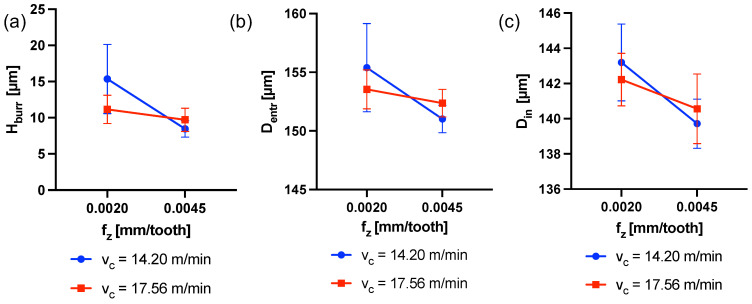
(**a**) Interaction plot of $f_{z}$ and $v_{c}$ on *H*_burr_. (**b**) Interaction plot of $f_{z}$ and $v_{c}$ on *D*_entr_. (**c**) Interaction plot of $f_{z}$ and $v_{c}$ on $D_{\mathrm{in}}$.

The TWA analysis results concerning *H*_burr_ as a function of the cutting parameters highlighted statistically relevant dependency of the response on $f_{z}$, $v_{c}$ and their interaction (*p*-Value ($f_{z}$) < 0.001, *p*-Value ($v_{c}$) = 0.027, *p*-Value ($f_{z}$$v_{c}$) < 0.001). Figure 16a shows how *H*_burr_ decreases as feed per tooth increases and how it varies according to the interaction between $f_{z}$ and $v_{c}$. This complex outcome to explain would need further investigation.

As for the diameters *D*_entr_ measured at the borehole entrance, the KW analysis revealed $f_{z}$ as the only affecting parameter (*p*-Value ($f_{z}$) < 0.001). In fact, an increase in the entrance diameter is observed when the feed per tooth is low. This is due to the increased time the tool spends inside the hole while rotating. Figure 16b shows the effect of cutting parameters on *D*_entr_.

**Figure 17:**
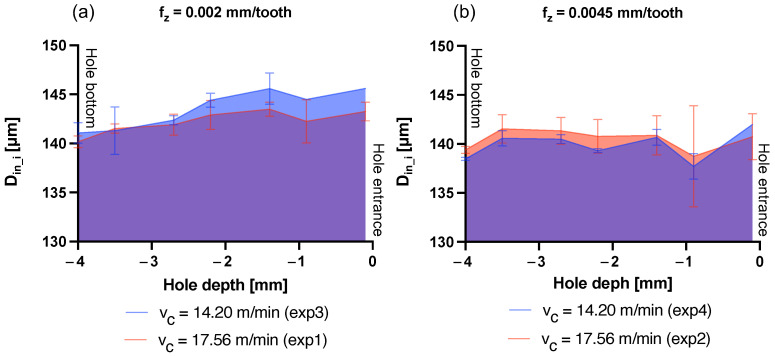
Inner hole *D*_in_i_ (i = 1–7) variation over hole depth. (**a**) *D*_in_i_ variation over the hole depth for exp1 and exp3. (**b**) *D*_in_i_ variation over the hole depth for exp2 and exp4.

**Figure 18:**
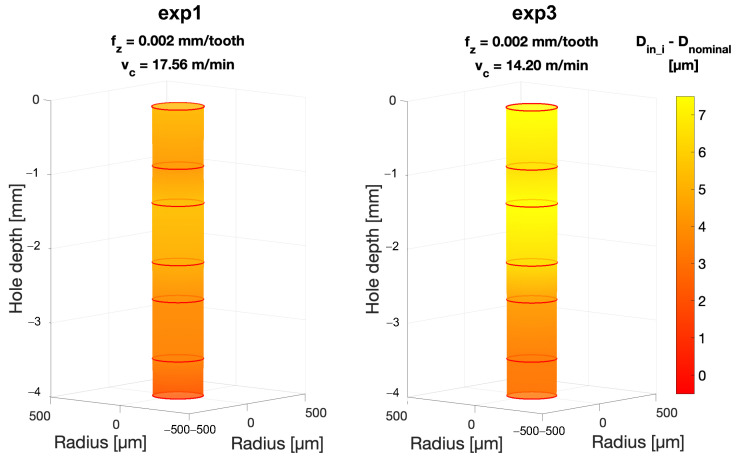
Inner hole diameter variation over the hole depth for exp1 and exp3 ($f_{z}$ = 0.002 mm/tooth) compared to the nominal $D_{\mathrm{nominal}}$. The color gradient is associated with the difference between the measured diameter *D*_in_i_ (i = 1–7) and $D_{\mathrm{nominal}}$.

**Figure 19:**
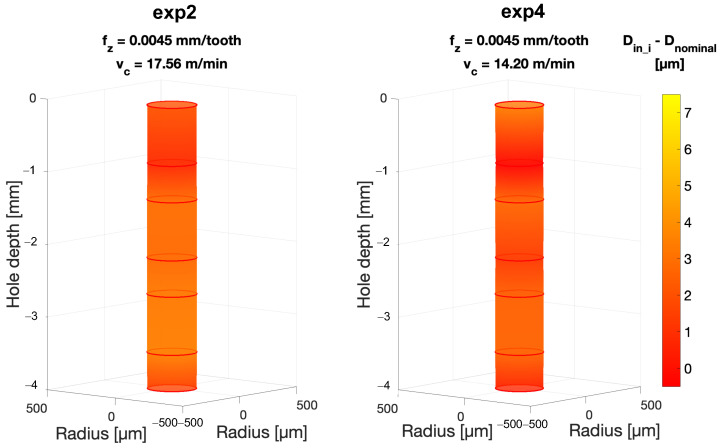
Inner hole diameter variation over the hole depth for exp2 and exp4 ($f_{z}$ = 0.0045 mm/tooth) compared to the nominal $D_{\mathrm{nominal}}$. The color gradient is associated with the difference between the measured diameter *D*_in_i_ (i = 1–7) and $D_{\mathrm{nominal}}$.

A similar result is obtained when considering the average inner diameter $D_{\mathrm{in}}$, considered as the average value of the measurement points *D*_in_1_–*D*_in_7_ taken for each hole. Also, in this case an influence of the cutting parameter $f_{z}$ is confirmed by the KW test (*p*-Value ($f_{z}$) < 0.001). In fact, as shown in Figure 16c, $D_{\mathrm{in}}$ decreases as $f_{z}$ increases. Our investigation also allows us to observe how the diameter of the hole is larger toward the inlet than toward the bottom. This can be observed in Figure 17, in which *D*_in_1_–*D*_in_7_ are plotted for the four experiments along the hole axis. Peck drilling involves periodically retracting the drill from the hole, which can lead to more wear at the top of the hole due to multiple drill insertions. This effect is also made clear by the graphs in Figure 18 and Figure 19. Here, the circumferences with diameters equal to *D*_in_i_ (i = 1–7) have been plotted in their respective measurement positions along the axis of the hole. The color gradient of the surfaces joining these circumferences and reconstructing the 3D geometry of the hole depends on the difference between the measured *D*_in_i_ and nominal diameters $D_{\mathrm{nominal}}$. The effect of hole enlargement is mainly observable in exp1 and exp3, where a cone shape can be detected (Figure 17a and Figure 18).

Table 5 shows a summary of the drilling parameters recommended based on the experimental results of thrust force and hole quality.

## 4. Discussion

In light of the limited literature on deep-hole microdrilling of magnesium, an investigation of the effect of feed per tooth and cutting speed on hole quality and thrust force was carried out in this study to obtain holes with a diameter equal to 0.138 mm and very high AR equal to about 36 on a pure Mg sample. As performance indexes for the evaluation, the maximum and average thrust force, $F_{z\_\max}$ and $F_{z\_\mathrm{avg}}$, respectively, were analyzed. Similarly, an investigation of hole quality in terms of burr height *H*_burr_, entrance diameter *D*_entr_, and diameter along the axis of the hole was conducted.

Concerning the $F_{z\_\max}$, the maximum thrust force for each run, no statistically relevant dependence with the factors was revealed from the TWA. This could be related to the nature of $F_{z\_\max}$. Indeed, Figure 14 shows variability of the peak values $F_{z\_\mathrm{peck}}$ with the peck number. It can be assumed that the significant variation in forces across consecutive drilling cycles indicates that the chip formation and motion within the flutes is a stochastic process [37]: $F_{z\_\mathrm{peck}}$ depends on random factors, such as the chip friction on the flute and hole wall and local lubrication conditions. The twist drill often fails to properly extract all the chips formed in the cutting area. As the chip clogs the hole and the drill is ready to start a new peck, more material is present than expected, causing a higher solicitation at the twist drill tip. Regarding the stability of the cut during the drilling operation, a few trends emerged. It is clear that exp2 ($f_{z}$ = 0.0045 mm/tooth, $v_{c}$ = 17.56 m/min) presents the most stable cut with an increase in the thrust force peaks in the last part of the drilling operation where the hole is deeper and consequently the chip evacuation is hindered (Figure 14b). The reason behind the stability of exp2 might reside in the combination of the thermal softening induced by the high levels of cutting speed and the quick chip extraction caused by the feed rate $v_{f}$ defined in Equation (2).

(2) $$v_{f}=n\cdot f_{z}\cdot Z$$

On the contrary, exp4 ($f_{z}$ = 0.0045 mm/tooth, $v_{c}$ = 14.20 m/min) presents the most unstable cut with an increase in the thrust force peaks early in the operation (Figure 14d). To have a better understanding of how the combination of the used parameters affects the maximum thrust force, further investigation would be necessary. For instance, a lower peck depth could be applied to the second half of the hole, in order to facilitate chip evacuation reducing the volume of the produced chip. Conversely, reducing the pecking depth could lead to lower drill bit stability, worse surface finish, and higher diametral, roundness, and cylindricity errors because of a higher number of tool re-engagements [38].

On the other hand, $F_{z\_\mathrm{avg}}$ has been shown to be influenced by both factors significantly. The obtained trends are in agreement with the theory of the chip formation process and the expected effect of the cutting parameters. Indeed, an increase in $f_{z}$ is associated to an increase in $F_{z\_\mathrm{avg}}$ due to the fact that the thrust the tool exerts on the target material increases [39,40]. In fact, the feed per tooth significantly influences chip thickness and thus the resulting cutting force. On the contrary, an increment of $v_{c}$ produces a decrease in $F_{z\_\mathrm{avg}}$, caused by the material softening induced by the increment of temperature in the cutting region. When the cutting speed is high, the strain rate is also high, leading to an increase in temperature within the cutting zone. This, in turn, induces thermal softening in the workpiece, leading to a lower force being required to remove the chip [41].

Typically, during the first stage of drilling, as the tool enters into the workpiece, plastic deformation occurs, whereby material in front of the chisel edge is pushed to the sides of the tool. As the drill penetrates deeper, the material deforms and flows plastically around the circumference of the hole, thus forming burrs [42,43,44]. In this study, the results concerning the burr formation on the hole entrance have demonstrated a dependence on the interaction between the two cutting parameters, but mainly on $f_{z}$. Although higher $v_{f}$ (i.e., *n* and/or $f_{z}$) typically increases the heat generated during the cutting process, which exacerbates burr formation due to thermal expansion and plastic deformation [43], this article observes a reduction in burr height as $f_{z}$ increases. This outcome could be linked to the dwell time inside the hole: lower feeds mean longer time and thus more defects at the entrance, high feeds mean shorter dwell time and thus smaller defects. Moreover, if it is assumed that the burr at the entrance of the hole is determined in the early stages of the whole deep-drilling process, it is possible to state that once the transient is completed, the burr height could be altered by the subsequent peck drilling steps. Although literature exists stating the contrary in other applications [26], we found a different result, to which we give this preliminary explanation that is not yet supported by experiments. In order to have experimental evidence, a specific campaign should be organized. These results were not observed in the preliminary study of deep-hole microdrilling conducted by the same authors of this paper on pure magnesium [27]. In that case the burr height produced by the 0.200 mm drill was shown to be dependent only on the cutting speed and not on the feed or their interaction. In light of this, we must take into consideration the dramatic impact of the size on the process at this scale. The effects of cutting tool size and, of course, cutting parameters, could be different depending on the scale and exhibit different behaviors [45].

Since the stiffness and twisting of a cutting tool bit are highly dependent on diameter and length, a thinner and longer bit is more susceptible to deformation and dynamic instabilities [46]. This aspect makes such tools prone to buckling that leads to sudden deformation under load, such as the curving of a column under compression. Consequently, it is acceptable that the deformation of the cutting tool while rotating, together with its runout, causes deformations to the hole’s walls as it advances into the material to be removed. Larger diameters are mainly observed at the borehole entrance, while diameters come closer to the nominal by going deeper: the borehole entrance is the most affected region by these effects [47]. This is reasonable because of the peck drilling strategy (i.e., several tool passages through the inlet of the hole), and it has been observed in these experiments. In light of this, if for higher feeds the thrust force increases, consequently the buckling tool curvature and thus the diameter enlargement should increase. However, the results reported here show the opposite of what would be expected: the entrance and inner diameters decrease by increasing the feed per tooth. This output, consequently, results from the sum of all the effects occurring during repeated tool passage. It is reasonable to assume that, increasing the dwell time of the tool inside the hole (i.e., with low feed per tooth) has a greater effect than increasing the thrust forces for this range of parameters.

To conclude, it must be noted that the most cylindrical shape is also associated with the most stable cutting condition in terms of thrust force, achieved in exp2 ($f_{z}$ = 0.0045 mm/tooth, $v_{c}$ = 17.56 m/min) (Figure). This might represent a first hint in the force–geometry correlation.

## 5. Conclusions

The following results have been achieved in this work.

- The effect of cutting parameters on thrust force were analyzed. The lowest values of thrust force were obtained for the highest level of cutting speed and the lowest level of feed per tooth.
- The hole with the best quality features is machined with the highest level of feed per tooth. Indeed, for this value, the lowest burr height and the most cylindrical shape of the hole were obtained. On the other hand, the interaction between $f_{z}$ and $v_{c}$ could affect the outputs in terms of hole quality.
- The hole entrance is the most affected region by the peck drilling strategy: the final effect on burr height and hole diameter comes from the sum of each tool pass between feed and retraction repeated several times. Moreover, a longer tool dwell time inside the hole results in increased defects.
- Hole maximum geometrical performance ensured by highest values of feed per tooth are however associated with the highest thrust forces and this is detrimental for the tool life. To balance this effect, cutting speed can be increased to induce material thermal softening and reduce the thrust force. Specifically, the best hole quality can be obtained with $f_{z}$ = 0.0045 mm/tooth, since this value results in short burrs and better cylindricity of the hole. Under this condition, it is advisable to use a high cutting speed of 17.56 m/min. Indeed, this condition lowers the thrust force values, providing lower drill bit loading, and thus more longevity.

The present study can aid in achieving precision manufacturing in the biomedical field, where the properties of the drilled holes in magnesium-based devices are crucial for drug delivery systems or other biomedical applications. By establishing a relationship between forces and hole morphology, refining machining parameters, enhancing process efficiency, and ensuring the desired geometric qualities of the drilled holes is possible, contributing to the overall success and reliability of magnesium-based microcomponents. Moreover, by establishing the relationship between forces and hole shape, monitoring can be performed.

## Figures and Tables

**Table 1:** Magnesium sample properties.

Composition	Hardness	Density
Mg-2.1Nd-0.2Zn-0.2Zr	52.6 HV (Load 300 g)	1.73 g/cm^3^

**Table 2:** Microtwist drills properties. $D_{\mathrm{tool}}$: tool diameter; $L_{\mathrm{flute}}$: flute length; $L_{u}$: tool usable length; ϵ: point angle; λ: helix angle; ψ: chisel edge angle; *r_e_*: cutting tool radius.

Tool ID	Function	$D_{\mathrm{tool}}$ [mm]	$L_{\mathrm{flute}}$ [mm]	$L_{u}$ [mm]	[∘]	[∘]	[∘]	*r_e_* [m]	Coating
342d0.14	PILOT	0.140	0.5	0.5	118	34	120	-	None
Custom	DRILL	0.138	1.1	5.5	120	24	130	2.599	None

**Table 3:** Cutting conditions. $D_{\mathrm{tool}}$: tool diameter; $Q_{\mathrm{peck}}$: peck depth; $Q_{\mathrm{hole}}$: blind hole depth; $f_{z}$: feed per tooth; *v_c_*: cutting speed; *n*: rotational speed.

Tool	$D_{\mathrm{tool}}$ [mm]	$Q_{\mathrm{peck}}$ [mm]	$Q_{\mathrm{hole}}$ [mm]	$f_{z}$ [mm/tooth]	$v_{c}$ [m/min]	*n* [rpm]
PILOT	0.140	0.10	0.4	0.0017	13	29,672
DRILL	0.138	0.07	5	0.002–0.0045	14.20–17.56	32,750–40,500

**Table 4:** Executed experimental drilling plan for the 0.138 mm holes.

Run Order	Experiment	$f_{z}$ [mm/tooth]	$v_{c}$ [m/min]
4	exp 1	0.0020	17.56
6	exp 1	0.0020	17.56
7	exp 1	0.0020	17.56
2	exp 2	0.0045	17.56
8	exp 2	0.0045	17.56
10	exp 2	0.0045	17.56
5	exp 3	0.0020	14.20
9	exp 3	0.0020	14.20
11	exp 3	0.0020	14.20
1	exp 4	0.0045	14.20
3	exp 4	0.0045	14.20
12	exp 4	0.0045	14.20

**Table 5:** Recommended drilling parameters based on the experimental results of thrust force and hole quality.

Parameter	Value	Outputs
$v_{c}$ [m/min]	17.56	Lower $F_{z\_\mathrm{avg}}$
$f_{z}$ [mm/tooth]	0.0045	Lower *H*_burr_
		Lower taper

## Data Availability

The original contributions presented in the study are included in the article, further inquiries can be directed to the corresponding author.

## Nomenclature

The following abbreviations are used in this manuscript:

ϵ	Tool point angle	[deg]
λ	Helix angle	[deg]
ψ	Chisel edge angle	[deg]
$D_{\mathrm{entr}}$	Hole entrance diameter	[µm]
$D_{\mathrm{in}}$	Inner hole diameter	[µm]
$D_{\mathrm{nominal}}$	Nominal hole diameter	[µm]
$D_{\mathrm{rod}}$	Sample diameter	[mm]
$D_{\mathrm{tool}}$	Tool diameter	[mm]
$\bar{d}$	Average grain size	[µm]
$f_{\mathrm{cutoff}\_\mathrm{drift}}$	Cutoff frequency for high-pass filter	[Hz]
$f_{\mathrm{cutoff}\_n}$	Cutoff frequency for low-pass filter	[Hz]
$F_{x}$	Cutting force in x direction	[N]
$F_{y}$	Cutting force in y direction	[N]
$F_{z}$	Cutting force in z direction	[N]
$F_{z\_\mathrm{avg}}$	Average cutting force in z direction	[N]
$F_{z\_\max}$	Maximum cutting force in z direction	[N]
$F_{z\_\mathrm{peck}}$	Maximum cutting force for each peck in z direction	[N]
$f_{z}$	Feed per tooth	[mm/tooth]
$H_{\mathrm{burr}}$	Average burr height	[µm]
$H_{\mathrm{burr}\_\mathrm{LH}}$	Burr height on the left of the burr profile	[µm]
$H_{\mathrm{burr}\_\mathrm{RH}}$	Burr height on the right of the burr profile	[µm]
$L_{\mathrm{flute}}$	Flute length	[mm]
$L_{u}$	Tool usable length	[mm]
*n*	Rotational speed	[rpm]
$Q_{\mathrm{hole}}$	Blind hole depth	[mm]
$Q_{\mathrm{peck}}$	Peck depth	[mm]
$r_{e}$	Radius of the tool cutting edge	[µm]
*th*	Sample thickness	[mm]
$v_{c}$	Cutting speed	[m/min]
$v_{f}$	Feed rate	[mm/min]
*Z*	Number of tool teeth	[-]
